# Plasma Tryptophan and the Kynurenine–Tryptophan Ratio Are Associated with the Acquisition of Statural Growth Deficits and Oral Vaccine Underperformance in Populations with Environmental Enteropathy

**DOI:** 10.4269/ajtmh.16-0037

**Published:** 2016-10-05

**Authors:** Margaret N. Kosek, Estomih Mduma, Peter S. Kosek, Gwenyth O. Lee, Erling Svensen, William K. Y. Pan, Maribel Paredes Olortegui, Jay H. Bream, Crystal Patil, Cesar Ramal Asayag, Graciela Meza Sanchez, Laura E. Caulfield, Jean Gratz, Pablo Peñataro Yori

**Affiliations:** 1 Department of International Health, Johns Hopkins School of Public Health, Baltimore, Maryland; 2 Global Health Research Center, Haydom Lutheran Hospital, Manyara, Tanzania; 3 Pain Consultants of Oregon, Eugene, Oregon; 4 Department of Global Community Health and Behavioral Sciences, Tulane University, New Orleans, Louisiana; 5 Haukeland University Hospital, Bergen, Norway; 6 Duke Global Health Institute, Nicholas School of Environment, Duke University, Durham, North Carolina; 7 Asociacion Benefica Proyectos de Informática, Salud, Medicina, y Agricultura (PRISMA), Iquitos, Peru; 8 Department of Molecular Microbiology and Immunology, Johns Hopkins Bloomberg School of Public Health, Baltimore, Maryland; 9 Department of Women, Children, and Family Health Science, University of Illinois at Chicago College of Nursing, Chicago, Illinois; 10 Universidad Científica del Peru, Iquitos, Peru; 11 School of Medicine, Universidad Nacional de la Amazonia Peruana, Iquitos, Peru; 12 Center for Global Health, University of Virginia, Charlottesville, Virginia

## Abstract

Early childhood enteric infections have adverse impacts on child growth and can inhibit normal mucosal responses to oral vaccines, two critical components of environmental enteropathy. To evaluate the role of indoleamine 2,3-dioxygenase 1 (IDO1) activity and its relationship with these outcomes, we measured tryptophan and the kynurenine–tryptophan ratio (KTR) in two longitudinal birth cohorts with a high prevalence of stunting. Children in rural Peru and Tanzania (*N* = 494) contributed 1,251 plasma samples at 3, 7, 15, and 24 months of age and monthly anthropometrics from 0 to 36 months of age. Tryptophan concentrations were directly associated with linear growth from 1 to 8 months after biomarker assessment. A 1-SD increase in tryptophan concentration was associated with a gain in length-for-age Z-score (LAZ) of 0.17 over the next 6 months in Peru (95% confidence interval [CI] = 0.11–0.23, *P* < 0.001) and a gain in LAZ of 0.13 Z-scores in Tanzania (95% CI = 0.03–0.22, *P* = 0.009). Vaccine responsiveness data were available for Peru only. An increase in kynurenine by 1 μM was associated with a 1.63 (95% CI = 1.13–2.34) increase in the odds of failure to poliovirus type 1, but there was no association with tetanus vaccine response. A KTR of 52 was 76% sensitive and 50% specific in predicting failure of response to serotype 1 of the oral polio vaccine. KTR was associated with systemic markers of inflammation, but also interleukin-10, supporting the association between IDO1 activity and immunotolerance. These results strongly suggest that the activity of IDO1 is implicated in the pathophysiology of environmental enteropathy, and demonstrates the utility of tryptophan and kynurenine as biomarkers for this syndrome, particularly in identifying those at risk for hyporesponsivity to oral vaccines.

## Introduction

Environmental enteropathy is a functional disorder of the gut resulting from multiple enteric infections which result in chronic intestinal immune activation, augmented intestinal permeability, and persistent systemic immune activation.^1^,^2^ In situations where there is limited access to improved sanitation and water sources, most children have some degree of the syndrome, with the level of disease activity within an individual varying over time.^3^,^4^ Environmental enteropathy is a likely and prominent cause of the observed failure of nutritional interventions to prevent or treat linear growth failure, even when done intensively under experimental, rather than programmatic conditions.^5^

In children living in poverty where numerous infections occur concurrently or in close succession,^6^ the orchestration of the immune response to these infections is likely to have an important influence on child growth and development. Although immune response may be adaptive and limit the infection, a vigorous immune response may also be nonadaptive, leading to local and systemic inflammation, dysregulation of the mucosal immune response, and metabolic changes that negatively influence child growth and development. Immunoregulatory signals act to influence amino acid and micronutrient uptake and nutrient utilization, so that metabolism is so closely linked to immune activity that some refer to these in singular as the immunometabolic system.^7^,^8^ In addition, attenuation of the immune response limits metabolic energy investment and tissue or organ damage incited by the immune response and may blunt the response to orally administered live vaccines.^9^

Clinical observations are substantiated by detailed observations of an attenuation of the immune responses to repeat dosing of lipopolysaccharide (LPS) or killed bacteria; also termed “endotoxin tolerance,” or immune desensitization or reprogramming. The latter two terms are more appropriate to the induced hyporeactivity, which are not just to LPSs but also to other bacterial response elements.^10^ Notably, the monocytes from patients surviving septic shock are resistant to in vitro activation for a period of at least 10 days.^11^ This demonstrates that this phenotype is stable for periods that could affect the reactivity and condition the response to separate discrete concurrent or nearly concurrent infections, especially in contexts where infectious exposures are intense or ongoing. We hypothesize that this state is analogous to the chronic low grade endotoxin exposure that exists in environmental enteropathy. Herein, we evaluated this hypothesis by assessing indoleamine 2,3-dioxygenase 1 (IDO1) activity via the measurement of plasma tryptophan and kynurenine concentrations and the tryptophan kynurenine ratio. As tryptophan is converted by IDO1 to kynurenine, low tryptophan, high kynurenine, and an elevated kynurenine–tryptophan ratio (KTR) indicate augmented IDO1 activity.

The KTR has been used as a marker of systemic inflammatory conditions including rheumatoid arthritis, inflammatory bowel disease (IBD), and acquired immunodeficiency syndrome. In Peru and Tanzania, we evaluated these markers, as well as citrulline, a biomarker of small intestinal functional mass and several key cytokines to determine whether they were associated with the progressive acquisition of deficits in statural growth in infancy and early childhood. In Peru, we additionally assessed whether these markers also predicted the other principal component of environmental enteropathy, failed mucosal immune response to vaccines.

## Materials and Methods

Child cohorts in Peru and Tanzania were enrollees of the etiology, risk factors, and interactions of enteric infections and malnutrition and the consequences for child health and development (MAL-ED) study.^12^–^14^ Newborns less than 17 days of age were enrolled if they lived in the recruitment zones in each area, were > 1,500 g at birth, had no evidence of congenital disease, were singletons, and had a mother who was at least 16 years of age. The research protocol was approved by the Institutional Review Board of Johns Hopkins Bloomberg School of Public Health, and the ethics committee of Asociacion Benefica PRISMA, the Regional Health Department of Loreto, Peru, the University of Virginia and the Institutional Review Board for Health Sciences Research, the National Institute for Medical Research of Tanzania, and the Ministry of Health and Social Welfare of Tanzania.

Children had monthly anthropometric assessments on the date of their birth and monthly thereafter with digital scales accurate to 0.01 kg and footboards and measured to the nearest 0.1 cm. Measurements were compared with the 2006 World Health Organization growth standards.^15^

Plasma samples were obtained at 3, 7, 15, and 24 months in Peru and at 7, 15, and 24 months in Tanzania for biomarker testing. As only Peru had 3-month samples, which represented a time point closely matched to the date of the primary immunization, only Peru data were included for the vaccine response analysis, whereas both sites contributed data to the growth analysis.

### Determination of citrulline, kynurenine, and tryptophan levels.

Standards for l-citrulline, l-tryptophan, and l-kynurenine (Sigma-Aldrich, St. Louis, MO) were prepared at 1 mg/mL in 10% (v/v) acetonitrile in water. Serial dilutions in water were completed to achieve a seven-point calibration set with ranges of citrulline (10–10,000 ng/mL), tryptophan (20–20,000 ng/mL, and kynurenine (1–1,000 ng/mL) in water. Quality control solutions were prepared at two levels by doping pooled human plasma with reference standards and stored at −80°C. Internal standard was prepared with l-citrulline-5-^13^C,5,5-d2 (Sigma-Aldrich), dl-tryptophan-d8 (CDW isotopes, Quebec, Canada) and l-kynurenine (ring-d4) (Buchem BV, Apeldoorn, The Netherlands) at a concentration of approximately 20% of the maximum calibrator for the respective analyte.

Calibrators, quality control, and samples were protein precipitated by vortexing 20 μL of plasma or calibrator with 50 μL of 0.2 molar trifluoroacetic acid containing the internal standards, adding 500 μL of acetonitrile, revortexing, and placing the vial in a −20°C freezer for 10 minutes. Samples were then centrifuged for 5 minutes at 5,000 g, and the supernatant was transferred to an autosampler vial for analysis.

Hydrophilic interaction liquid chromatography was performed on a Shimadzu LC-20 HPLC system (Columbia, MD) using a Cortecs HILIC 2.7 um 2.1 × 50 mm column (Waters, Milford, MA) at 40°C. Mobile phase A contained 50% (v/v) acetonitrile in water, mobile phase B contained 95% (v/v) acetonitrile in water, and both phases contained 12.5 mM formic acid. At a total flow of 0.75 mL/minute and column stabilization for 2.5 minutes at 92% A, a 5-μL sample was introduced, and a linear gradient to 1% A was achieved at 2.5 minutes and was held for 0.5 minute. A Sciex QTRAP 5500 tandem mass spectrometer (Framingham, MA) was programmed to the transitions detailed in Table 1, with a 600°C ionization temperature and 2,000 V ionization voltage in positive ionization mode.

#### Lactulose mannitol test.

Children were fasted (with the exception of breastmilk) for 2 hours before and 30 minutes after the administration of the disaccharide solution. Children were also encouraged to void before the administration of the solution. Lactulose was administered at 250 mg/mL and mannitol at 50 mg/mL at a dose of 2 mL/kg to a maximum administered dose of 20 mL as detailed in Kosek and others.^1^ Urine was collected for 5 hours into sterile containers with fixative and then frozen at −80°C until analysis. Concentrations of analytes were measured by high-performance liquid chromatography and pulsed amperometric detection in Tanzania^16^ or tandem mass spectrometry in Peru.^17^

#### Plasma cytokines.

Plasma cytokines were measured in Peru samples only using the Ultrasensitive Human Proinflammatory 9-plex assay from Meso Scale Discovery (MSD), Gaithersburg, MD. The MSD multispot array was run according to the manufacturer's protocol with minor modifications.^18^ All samples were run in duplicate. In brief, plates were preincubated with 25 μl of supplied diluents for 30 minutes, with shaking, at room temperature. Calibration curves were prepared in the diluents and ranged from 2,500 pg/mL to 0.15 pg/mL. After the 30-minute incubation period, 25 μl of plasma sample or calibrator was added to the wells. Plates were then incubated at room temperature for 2 hours with shaking. Plates were then washed with phosphate-buffered saline (PBS) and 0.05% Tween and then incubated with 25 μl of detection antibody for 2 hours at room temperature with shaking. After washing plates with PBS and 0.05% Tween, 150 μl of detection antibody was added. Plates were read using the MS2400 imager (MSD). The lowest limit of quantification (LLOQ) was defined as the lowest calibrator value at which the coefficient of variance of concentration was less than 25% and recovery of calibrator was within 25% of the expected value. All cytokine values that were below the LLOQ were considered undetectable and assigned a value equal to the plate-specific LLOQ for chemoluminescent detection (Meso Scale Development, Rockville, MD). C-reactive protein (CRP) was measured using Luminex technology (Myriad Rule Based Medicine, Austin, TX). Alpha-1-acid glycoprotein (AGP) was measured by radioimmune diffusion by previously specified methods.^19^

#### Statistical methods.

Individual biomarker concentrations were evaluated for association with weight-for-age Z-score (WAZ), length-for-age Z-score (LAZ), and weight-for-length Z-score at the age they were obtained. Linear regression models adjusting for age, gender, and nutritional status (LAZ or WAZ) at baseline, and including a child-level random effect to account for the fact that each child contributed multiple biomarker assessments to the analysis, were used to estimate associations between each individual biomarker (expressed per standard deviation [SD]) and changes in LAZ over subsequent periods in a month-by-month manner (i.e., t + 1, t + 2, t + 3…t + 10). These models were first fitted separately for Peru and Tanzania, and then combined models were built. This method also allows for the visual display of the dynamics of linear growth response associated with the biomarkers. The model assumed children with missing data was random and model fit was evaluated with partition of variance estimates. Because four biomarkers were tested (citrulline, kynurenine, tryptophan, and the KTR), *P* values were considered significant at the ≤ 0.0125 Bonferroni-corrected level.

To measure the ability of each candidate biomarker to predict impaired vaccine immune response, the plasma concentration of the biomarkers of interest were assessed in the 3-month samples and evaluated for their associations with the 7-month plasma sample for oral polio vaccine (OPV) response to all three serotypes via neutralization assay. IgG to tetanus, an intramuscular vaccine with no expected response impairment related to the level of disease activity of environmental enteropathy was also evaluated. Poliovirus serum neutralizing assays were performed by the Centers for Disease Control and Prevention in Atlanta, GA, in accordance with World Health Organization assays for all three serotypes.^20^

Immunization histories were captured on monthly visits where parents were interviewed and vaccine cards were reviewed. Trivalent OPV was administered at 2, 4, and 6 months, and differences in reports and vaccine cards were evaluated by repeated interview with family members and the local health center with resolution of differences favoring the report of the health-care center. Tetanus toxoid was administered at 2, 4, and 6 months as the pentalavent vaccine (diphtheria, tetanus, pertussis [DTP], *Haemophilus influenzae* B [Hib], and hepatitis B [HBV]).

Vaccine response as an outcome was measured as a dichotomous response. For the dichotomous response, vaccine failure was defined for OPV as log_2_[titer] < 3 and tetanus as < 0.1 IU/mL.^21^ The relationship between vaccine response with tryptophan, kynurenine, citrulline (all measured as μmol/L), and the KTR were evaluated using logistic regression models that controlled for LAZ at the time of biomarker assessment (3 months) as well as breastfeeding (categorized as exclusive, mixed, and weaned), number of days of antibiotic use up to the date of primary immunization, and diarrhea 3 days prior or 3 days after the primary vaccine administration at 2 months of age. Significant relationships were expressed as the increase in the odds of failure per change in biomarker concentration and as the odds of failure given a 1-SD change in the biomarker level to express the amount of variability in the population. Analyses were conducted on children who received three or more OPV doses by the 7-month blood draw. Goodness of fit was evaluated using the Hosmer–Lemeshow test. A receiving operator curve for KT and OPV1 vaccine failure was constructed to determine the optimal cutpoint to guide in decision-making of this assay when used as a predictive biomarker of vaccine failure.

## Results

There were 303 children enrolled at the Peru site, 275 of which had at least one tryptophan, kynurenine, and citrulline measurement, and 198 children who were retained at 24 months of age. In Tanzania, 262 children were enrolled, 219 of which had at least one tryptophan, kynurenine, and citrulline assessment, with 211 children retained throughout the 24-month study period. Baseline anthropometry and characteristics of children and their incidence of infectious diseases are compared in Table 1, and demonstrate similar breastfeeding patterns and incidence of acute lower respiratory infections, but rates of diarrheal disease were greater in Peru than in Tanzania. Human immunodeficiency virus (HIV) testing was not done in this study, but rates of HIV in this area of Peru and in Tanzania are known to be low, and it is unlikely that any participants in either site were HIV positive.^13^,^14^,^22^ Systematic screening for malaria parasitemia was not done as part of the study protocol, and diagnosis was done only in febrile children. Both areas are hypoendemic for malaria transmission. In Peru, *Plasmodium vivax* predominates, and in Tanzania, malaria is only due to *Plasmodium falciparum*.

Children with biomarker assessment did not differ by birthweight, LAZ at 1 month of age, maternal age, maternal education, or per capita income from children who did not contribute samples.

### Biomarkers.

Assays were available for 1,251 samples from 494 children. Mean plasma citrulline concentrations were 21.1 μM (95% confidence interval [CI] = 12.7–32.8) (Table 2). Citrulline was not associated with LAZ, WAZ, or weight-for-height Z-score at the time of biomarker assessment, except in children 3 months of age, where citrulline was negatively correlated with LAZ (rho = −0.16, *P* = 0.01).

Mean concentrations of kynurenine, tryptophan, and the KTR were 2.9 μM (95% CI = 1.8–4.5), 49.9 μM (95% CI = 22.1–74.9), and 60.6 μM (95% CI = 38.1–121.8) across sites and age groups (Table 2). Tryptophan and kynurenine levels declined with age in both Peru and Tanzania, whereas the KTR declined in Peru but increased in Tanzania. The relationship between LAZ and WAZ at the time of biomarker assessment and kynurenine measures differed between children in Peru and Tanzania. In Peru, low kynurenine concentrations were associated with improved LAZ in children at 3 months of age only (rho = −0.16, *P* = 0.03), but at 7, 15, and 24 months there were no significant associations detected between kynurenine and anthropometric indices, nor were there any associations with concurrent anthropometric indices and tryptophan or KTRs. In Tanzania, however, there were moderate inverse associations between the KTR and LAZ and WAZ at 7, 15, and 24 months (Spearman's ρ for LAZ = −0.34 [−0.42 to −0.25, *P* < 0.0001] and WAZ = −0.25 [−0.34 to 0.16, *P* < 0.0001]). These associations were driven by differences in tryptophan, but the association was stronger with the ratio than with the individual components.

Because high lactulose recovery reflects permeability to abnormally large molecules, it is often interpreted to express a greater risk of microbial translocation and systemic immune activation in environmental enteropathy. Levels of kynurenine and tryptophan were not related to lactulose permeability, whereas children with low mannitol excretion defined as less than 90% of the population were likely to have higher tryptophan (55.1 versus 51.1 μM, *P* = 0.05) and kyneurenine levels (3.4 versus 3.1 μM, *P* = 0.02). Children with lower citrulline levels were more likely to have increased permeability to lactulose as assessed by a lactulose excretion greater than or equal to 90% of the population (19.1 versus 22.2 μM, *P* < 0.01) but citrulline levels were not associated with mannitol excretion.

#### Cytokines and systemic inflammatory markers.

AGP was measured in 436 samples from 219 children in Tanzania and 578 samples from 254 children in Peru. Mean levels were 113 (95% CI = 73–177) and did not differ by age or country. Cytokine assays (567 samples from 254 children) and CRP (578 samples from 256 children) were available from children in Peru only. Interferon-γ (IFN-γ) mean levels were 3.08 pg/mL (95% CI = 1.45–12.18), mean interleukin (IL)-10 levels were 5.53 (95% CI = 3.25–10.82), and IL-6 levels were 1.28 (95% CI = 0.62–4.61). The mean CRP was 1.05 mg/L (95% CI = 0.14–12.0). Correlations were examined, and across the different age groups, the coefficients were highest between CRP and AGP (95% CI = 0.54–0.68, *P* < 0.01) and were similar across the age groups (Table 3). Correlations between IL-6 and CRP were of similar magnitude (Table 3). The KTR had a strong correlation with IFN-γ (0.24–0.39, *P* < 0.01) and IL-10 (0.27–0.38, *P* < 0.01) across age groups and significant, but lower correlations with CRP, AGP, and IL-6 (Table 3).

### Statural growth.

**Figure 1.**
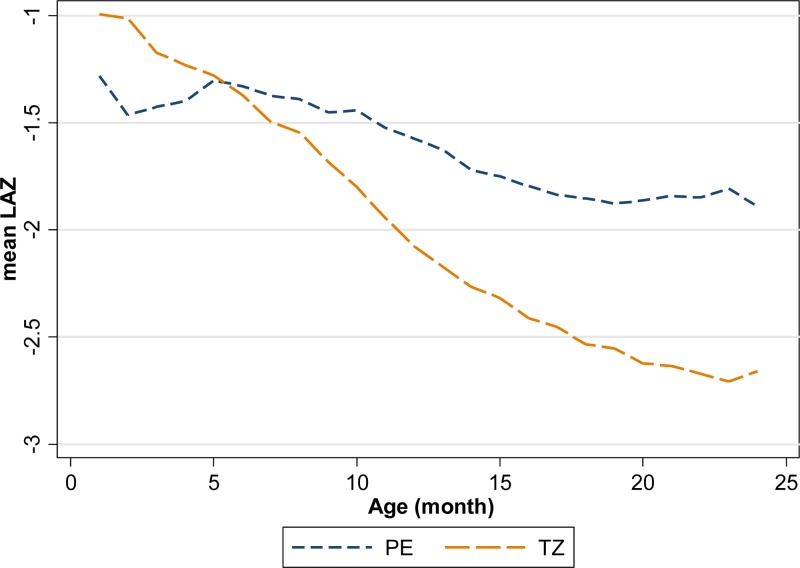
Observed length-for-age Z-scores of children in Peru and Tanzania demonstrate progressive linear growth failure.

The mean LAZ in Peru at 1 month of age was −1.3 and decreased to −1.9 at 24 months (Figure 1

). At 24 months, 39.9% of children in Peru were stunted. In Tanzania, the mean LAZ at 1 month of age was −1.0 and decreased to −2.7 at 24 months at which time 71.3% of the children were stunted.

**Figure 2.**
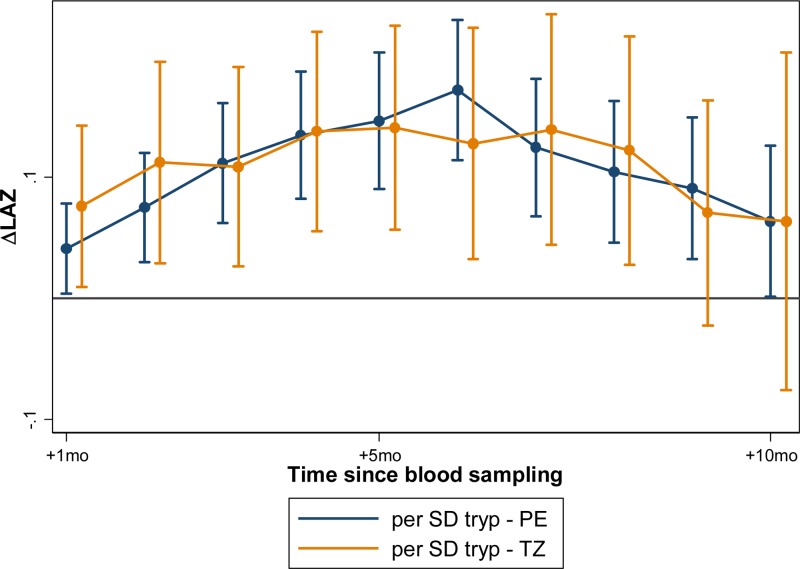
Tryptophan concentrations are associated with subsequent improved linear growth in children in Peru and Tanzania when measured at 3, 7, 15, and 24 months of age. Children in Peru had consistently improved linear growth for 10 months after the assessment at these four time points, whereas children in Tanzania exhibited improved growth for 8 months after the assessment.

**Figure 3.**
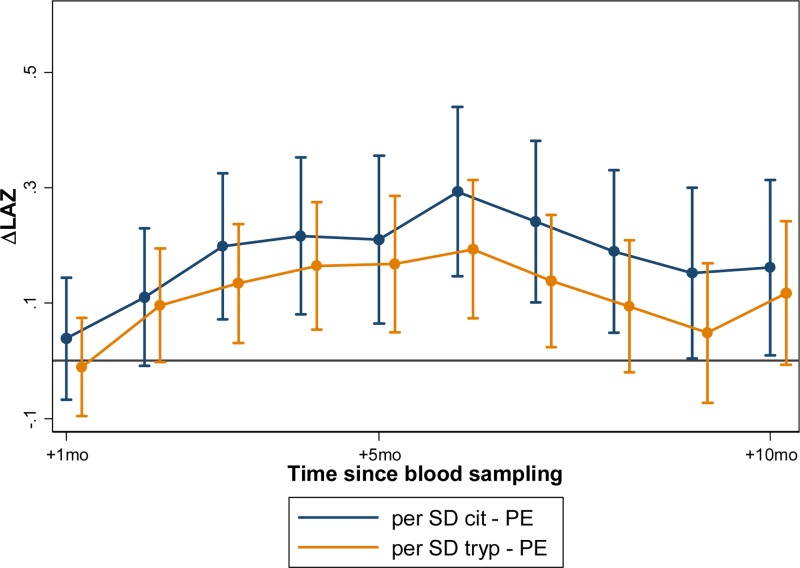
Citrulline and tryptophan concentrations when measured at 3 months of age are associated with linear growth between 3 and 8 months (citrulline) and 3 and 7 months (tryptophan) subsequent to their assessment in Peruvian children. The association between citrulline and subsequent statural growth was only present in children when measured at this young age, whereas the strength of associations between tryptophan and subsequent statural growth were greatest in this age group, but persisted through infancy and early childhood (see Figure 2).

In models adjusting for baseline LAZ, age, and gender, significant associations were noted in linear growth after biomarker assessment in both Peru and Tanzania. These effects were similar in magnitude when measured at 7, 15, and 24 months (Table 4). In Peru, greater linear growth was observed from 1 to 10 months after biomarker assessment, whereas in Tanzania, the association was seen from 1 to 8 months after biomarker assessment. In both countries, the strength of association increased monthly to the time point 6 months after the biomarker assessment, and then tapered from that point forward (Figure 2

). At this time of peak effect size when measured at 7, 15, and 14 months of age, every 1-SD increase in tryptophan was associated with a gain in LAZ of 0.10 over the next 6 months in Peru (95% CI = 0.05–0.16, *P* < 0.001); and a gain in LAZ of 0.13 over the next 6 months in Tanzania (95% CI = 0.03–0.22, *P* = 0.009). Tanzanian children whose tryptophan was at the 90th percentile gained an estimated 0.29 (95% CI = 0.07–0.52, *P* = 0.009) in LAZ over the 6 months after the measurement than those at the 10th percentile; whereas Peruvian children whose tryptophan was at the 90th percentile gained an estimated 0.24 LAZ more than those whose tryptophan was at the 10th percentile (95% CI = 0.26–0.52, *P* < 0.001). In Peru, where tryptophan was additionally measured at 3 months, the association with growth was significantly stronger, and a 1-SD increase in tryptophan was associated with a 0.36 increase in change in LAZ over the subsequent 6-month period (95% CI = 0.18–0.54, *P* < 0.001) (Figure 3

). Neither kynurenine nor the KTR demonstrated significant relationships with growth in similar models.

In models adjusting for baseline LAZ, age, and sex, citrulline was significantly associated with growth in Peru when measured at 3 months, but not at 7, 15, or 24 months of age. At 3 months, every 1-SD increase in citrulline concentration was associated with a gain in LAZ of 0.19 over the next 6 months in Peru (95% CI = 0.07–0.31, *P* = 0.002); or a difference in 0.50 Z-scores over 6 months between children at the 90th percentile versus the 10th percentile for citrulline (95% CI = 0.19–0.81, *P* = 0.002; Figure 3). In Tanzania, citrulline concentration was not associated with growth.

### Vaccine response.

In Peru, vaccine response was available for 256 children, but 43 children received fewer than three OPV doses before the 7-month blood draw and were excluded. Among these, 173 provided a blood sample at 3 months for biomarker assessment, which was a protocol addition that began on November 10, 2010. After that date, specimens were obtained from 94% of all newly enrolled subjects and were available in 67.6% (*N* = 173) of the entire Peruvian cohort.

**Figure 4.**
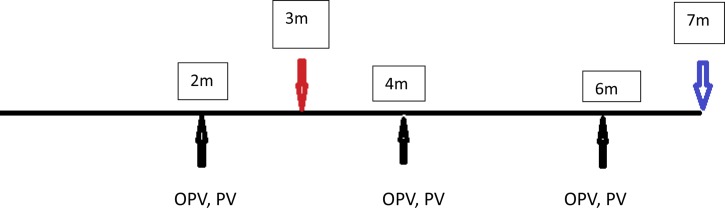
Tryptophan and kynurenine were assessed at 3 months, and antibody response was evaluated at 7 months. Trivalent oral polio vaccine (OPV) and the pentavalent vaccine were administered concurrently at 2, 4, and 6 months in Peru.

Trivalent OPV was administered at 2, 4, and 6 months of age. No inactivated polio vaccine (IPV) was administered in this population. Among the children who received at least three OPV doses before 7 months of age, when vaccine responsiveness was measured, 80.9% had completed the correct number of doses of tetanus toxoid administered as pentavalent vaccine (HBV, HiB, DPT) (Figure 4

).

Nonresponse to at least one of the poliovirus serotypes was observed in 22.8% of children. Nonresponsiveness was most common to serotype 3 (18.7%), whereas 7.0% and 2.3% failed to respond to poliovirus serotype 1 and 2, respectively. Failure to respond to tetanus was seen in only 4.6% of children (1.4% if children received all recommended doses). When a KT value of 52 is used as the cutoff value, OPV1 vaccine failure is predicted with 76% sensitivity and 50% specificity.

Citrulline and tryptophan concentrations were not associated with vaccine failure to any of the antigens evaluated. Kynurenine concentration, however, was associated with failure to respond to OPV serotype 1. After controlling for LAZ at 3 months of age, breastfeeding status, the number of days of exposure to antibiotics and diarrhea before 2 months of age, a 1-μM increase in kynurenine was associated with a 1.63 times higher odds of OPV1 vaccine failure (95% CI = 1.13–2.34) and 1-SD increase in kynurenine concentration was associated with a 1.90 (95% CI = 1.18–3.10) increase in the odds of failure to serotype 1. Similarly, a 1-SD increase in the KTR increased the odds of vaccine failure to OPV serotype 1 by 1.89 (95% CI = 1.21–2.97). No biomarkers were associated with response to tetanus toxoid.

## Discussion

In the present study, we find plasma concentrations of tryptophan to be associated with the subsequent acquisition of statural growth deficits in children living in poverty in Tanzania and Peru. Stunting is currently the best surrogate available for the loss of human potential of children in resource-constrained environments, and it has now clearly been demonstrated to be linked with deficiencies in reading and math achievement after controlling for years of schooling in income and grade level in multiple contexts and in adult-income productivity.^23^–^26^ Identifying mechanistic pathways associated with these physiologic insults is important to the planning of their treatment and mitigation. The effect size of this association is relatively large, so that in Peru, the difference in linear growth between 6 and 12 months in a child with a tryptophan level increased by 1-SD at 6 months would be augmented by 1 cm, which compares well to other early candidate biomarkers of environmental enteropathy.^27^ In addition, we identify the KTR as a predictive biomarker for vaccine failure to wild poliovirus type 1 (WPV1).

The results for citrulline demonstrates a limited association with acquired linear growth deficits when measured at 3 months, but no association with subsequent linear deficits when measured at 7, 15, and 24 months of age. The underlying reason for this is not clear, and in comparable studies in populations of nonstunted children in Tanzania, citrulline concentrations at 6 weeks were not associated with the time to first stunting event,^28^ although the same group demonstrated a positive correlation between plasma citrulline and change in WAZ over a 3-week period after zinc supplementation among children 6–23 months of age in Burkina Faso.^29^ Although the analyses are notably different, the effect size of the association seen in this study in the range of 0.2–0.3 LAZ over a period of 2–10 months after assessment at 3 months of age is large and compares favorably to any previously described biomarker for environmental enteropathy in this regard. As this is a critical period of rapid growth, the use of this biomarker may be considered in early intervention studies (with antibiotics, microbial/probiotic therapy, food or zinc supplements), whereas the utility of this biomarker in older children does not appear to have value. Citrulline concentrations were inversely associated with measures of systemic inflammation (CRP, AGP, and IL-6) at all ages, a finding that is not seen in studies of short bowel syndrome or IBD, but is consistent with current thinking that enterocyte function, intestinal infection and systemic immune activation are closely linked in environmental enteropathy.

The study further demonstrates the association of the KTR and OPV underperformance. There are three wild poliovirus types (type 1, type 2, and type 3). As wild poliovirus type 2 (WPV2) was last reported in 1999, and WPV3 in 2012,^30^ the identification of a biomarker that identifies the risk of vaccine failure to the remaining circulating strain of WPV1 is of particular importance for disease elimination strategies. It might also be relevant rotavirus, another oral vaccine that has impaired efficacy in settings where environmental enteropathy is highly endemic. Fecal reg1B has been previously demonstrated to be associated with impaired response to OPV2 and OPV3, and fecal calprotectin to OPV3.^2^ Screening populations with high concentrations of biomarkers, who may benefit from alternatives to the routinely recommended trivalent OPV, such as the targeted use of monovalent or bivalent OPV or IPV in populations at highest risk of vaccine underperformance could be of utility. It should also be noted that the failure of select children to respond to live oral vaccines is a simple surrogate of failed response to pathogen challenge that children living in settings of extreme poverty are likely to experience throughout their early childhood. Interventions that target children with this impaired mucosal response to ingested pathogens are likely to be of increased importance in the prevention or attenuation of adverse health outcomes in settings where pathogen exposure is intense and continuous. Enriching intervention trials done with antibiotics, probiotics, or other select interventions such as those to treat^31^ enteric protozoa have enhanced benefit in this subset of children that are more likely to have a diminished ability to respond to mucosal pathogen challenges.

Tryptophan can be catabolized by IDO1 or tryptophan 2,3-dioxygenase (constitutively expressed in the liver), but the catabolism is principally regulated in most settings by IDO1 which is induced by and regulated by the immune system. IDO1 is heavily expressed in the intestine^32^ and inducible upon intestinal inflammation in Crohn's disease and in HIV.^33^ The activity of this enzyme results in the conversion of tryptophan to l-kynurenine. Kynurenine activates the aryl hydrocarbon receptor and promotes Treg development. In the intestine specifically, AhR ligands promote local IL-22 and IL-17 production by innate lymphoid cells which have been shown to regulate mucosal reactivity.^34^,^35^

The measurement of IDO1 activity has been used to predict disease progression in HIV in which it is hypothesized that by inducing the loss of Th17 cells and upregulating Treg activity. IDO1 activity (denoted by depressed plasma tryptophan and elevated tryptophan metabolites) facilitates microbial translocation and systemic immune activation.^35^ We hypothesize that a similar process occurs in children with environmental enteropathy. The strength of association between plasma concentrations of tryptophan and the l-KTR and linear growth is stronger than that of CRP, AGP, and IL-6 suggesting that it is, in the case of environmental enteropathy, a more specific and predictive prognostic marker than these more common markers of systemic inflammation. Furthermore, the association of the KTR with IL-10 is both strong and consistent across age groups, which supports the putative role of IDO1 induction as one of immunotolerance, rather than just the expression of an alternative marker of systemic immune activation. This pattern is the pattern that one would predict as one resulting from IDO1 activity and kynurenine-based activation of T regs,^8^ and is interesting given the purported immunomodulatory role of IL-10 in intestinal injury and inflammation.^36^

Tryptophan concentrations are also influenced by dietary intake. In Tanzania, maize is the staple, and low tryptophan intakes have been documented. Symptoms of tryptophan deficiency may occur with intakes as little as 25% below the requirement, and include anorexia and impaired growth.^37^ Clinical findings of pellagra were not noted in children in the study cohort, but this does not exclude the possibility that deficiency in this amino acid and niacin did not play a role in the dramatic acquired postnatal linear growth deficits seen in this population, as the lower values here below 20 are far from the reported age normal values of 55 in these children.^38^ In this population, supplementation or dietary diversification may have an important role in optimizing early child growth as may the use of modified staples such as quality protein maize,^39^ which has high concentrations of lysine and tryptophan.^40^ Alternatively, intestinal microbiota may also have important roles in the formation of tryptophan and metabolites.^34^ Common components of the microbiome produce tryptophan and its metabolites, and germ-free animals have lower plasma tryptophan concentrations than conventionally fed or humanized mice.^41^

As the relative contributions of dietary intake, host microbiome, and host IDO1 activity in the genesis of the observed low levels of tryptophan are unclear, so is the mechanism by which low tryptophan levels influence growth. Recently, it has been shown that piglets with select dietary amino acid deficiencies (limited tryptophan, threonine, methionine, and cysteine) reduce the utilization of amino acids for growth after a period of poor health.^42^ Prior studies in growing pigs demonstrate that in conditions of deteriorated housing and moderate systemic inflammation—highly analogous to environmental enteropathy—tryptophan metabolism is modified and not efficiently used for protein deposition.^43^,^44^ Whether these growth insults are due to the combined effects of direct competition between aromatic amino acids diverted from protein deposition to immune response or due to the oxidation of unbalanced amino acids remains to be clearly demonstrated.^45^

IDO1 is stimulated by IFN-γ, LPS, and agonists of toll-like receptors (TLRs). IDO1 activity has been shown to alter the balance of Th17 to regulatory (Treg) cells. Th17 cells are purported to have a key role in mucosal defense, and their loss is hypothesized to be a key step in the augmented permeability, bacterial translocation, and systemic inflammation that is characteristic of progressive HIV disease. The activation of Treg cells has immunosuppressive effects, and kynurenine is a ligand for aryl hydrocarbon receptor, a key factor in the maintenance of an endotoxin-tolerant state. In rectosigmoid biopsies in patients with HIV, the loss of Th17 and increase in Treg cells were associated with an increased rate of disease progression. We hypothesize that this manifestation of immunotolerance at the level of the intestinal mucosa has important effects in modulating the host response to subsequent infections, including, but not solely as observed in response to the OPV. Optimizing early child growth is a major goal to diminish child mortality and morbidity in children living in poverty, and the control of infectious diseases and systemic immune activation associated with growth failure have an important role in achieving this goal. Similarly, addressing the limited efficacy of oral vaccines in certain epidemiological settings is an area of importance, as these vaccines fail largely in the most vulnerable and isolated populations that would most benefit from their protection. Herein, we describe a simple assay that allows for the identification of populations at risk of these phenomenons related to environmental enteropathy that should allow trials of interventions to be enriched to include those at greatest risk for these outcomes.

## Figures and Tables

**Table 1:** Baseline characteristics of study populations in Peru and Tanzania

	Peru Mean (SD)	Tanzania Mean (SD)
Mean enrollment weight	3.094 (0.026)	3.372 (0.029)
LAZ at 1 month	−1.274 (0.060)	−1.024 (0.069)
Duration of exclusive breastfeeding, days (median, 10th percentile, 90th percentile)	19 (3–120)	34 (10–87)
Age at weaning, days (median, 10th percentile, 90th percentile)	576 (435–784)	545 (357–736)
Diarrhea episodes in the 1st year of life	4.16 (3.20)	1.75 (1.64)
Diarrhea episodes in the 2nd year of life	4.54 (3.05)	0.89 (1.19)
ALRI episodes in 1st year of life	0.46 (0.82)	0.24 (0.48)
ALRI episodes in 2nd year of life	0.47 (0.78)	0.41 (0.57)

ALRI = acute lower respiratory infections; LAZ = length-for-age Z-score; SD = standard deviation.

**Table 2:** Kynurenine and tryptophan mean concentrations (shown with 95% CI) measured in plasma from 494 unique children and 1,251 samples in Peru and Tanzania

	*N*	Citrulline (μM)	Kynurenine (μM)	Tryptophan (μM)	KTR (ratio * 1,000)
Peru					
3 months	189	20.2 (13.2–34.3)	3.7 (2.7–5.9)	72.9 (52.4–93.0)	52.8 (36.3–81.6)
7 months	230	14.5 (9.6–21.8)	3.0 (2.2–4.3)	56.3 (40.0–68.8)	53.9 (38.1–85.1)
15 months	218	20.3 (13.6–29.3)	2.6 (1.8–4.1)	48.1 (28.8–70.2)	54.0 (36.4–92.8)
24 months	178	28.7 (19.9–42.0)	2.0 (1.8–4.5)	46.2 (25.9–67.1)	44.7 (30.3–77.3)
Tanzania					
7 months	124	18.7 (11.9–27.4)	3.4 (2.5–4.9)	45.4 (24.8–65.1)	79.9 (51.6–137.6)
15 months	152	22.2 (15.6–31.2)	3.1 (1.9–4.5)	36.9 (14.9–57.3)	88.6 (53.9–163.4)
24 months	160	24.3 (17.5–35.8)	2.7 (1.5–4.2)	28.5 (11.5–56.3)	94.5 (55.8–204.5)
Total	1,251	21.1 (12.7–32.8)	2.9 (1.8–4.5)	49.9 (22.1–74.9)	60.6 (38.1–121.8)

CI = confidence interval; KTR = kynurenine–tryptophan ratio.

**Table 3:** The correlation between cytokines, systemic markers of inflammation associated with endotoxin tolerance, and citrulline in Peruvian children

	IFN-γ	IL-10	IL-6	AGP	CRP
7 months (*N* = 198 [cytokines]; *N* = 209 [AGP]; *N* = 199 [CRP])					
Citrulline	−0.03 (*P* = 0.69)	−0.07 (*P* = 0.32)	−0.17 (*P* = 0.01)	−0.23 (*P* = 0.23)	−0.21 (*P* ≤ 0.01)
Tryptophan	−0.16 (*P* = 0.02)	−0.12 (*P* = 0.09)	−0.17 (*P* = 0.01)	−0.24 (*P* < 0.01)	−0.23 (*P* < 0.01)
KTR	0.35 (*P* < 0.01)	0.36 (*P* < 0.01)	0.26 (*P* < 0.01)	0.16 (*P* = 0.02)	0.16 (*P* = 0.02)
AGP	0.15 (*P* = 0.03)	0.03 (*P* = 0.71)	0.36 (*P* = 0.01)	–	0.54 (*P* < 0.01)
CRP	0.31 (*P* < 0.01)	0.16 (*P* = 0.03)	0.64 (*P* = 0.01)	0.54 (*P* < 0.01)	–
15 months(*N* = 192 [cytokines]; *N* = 208 [AGP]; *N* = 202 [CRP])					
Citrulline)	−0.16 (*P* = 0.03)	0.01 (*P* = 0.91)	−0.19 (*P* < 0.01)	−0.25 (*P* < 0.01)	−0.27 (*P* < 0.01)
Tryptophan	−0.16 (*P* = 0.03)	−0.15 (*P* = 0.04)	−0.21 (*P* < 0.01)	−0.27 (*P* < 0.01)	−0.27 (*P* < 0.01)
KTR	0.39 (*P* < 0.01)	0.38 (*P* < 0.01)	0.33 (*P* < 0.01)	0.27 (*P* < 0.01)	0.03 (*P* < 0.01)
AGP	0.17 (*P* = 0.02)	0.10 (*P* < 0.17)	0.41 (*P* < 0.01)	–	0.58 (*P* < 0.01)
CRP	0.25 (*P* < 0.01)	0.22 (*P* < 0.01)	0.50 (*P* < 0.01)	0.58 (*P* < 0.01)	–
24 months (*N* = 177 [cytokines]; *N* = 161 [AGP]; *N* = 177 [CRP])					
Citrulline	−0.06 (*P* = 0.40)	−0.15 (*P* = 0.04)	−0.27 (*P* < 0.01)	−0.21 (*P* = 0.01)	−0.34 (*P* < 0.01)
Tryptophan	−0.13 (*P* = 0.08)	−0.13 (*P* = 0.10)	−0.27 (*P* < 0.01)	−0.27 (*P* < 0.01)	−0.26 (*P* < 0.01)
KTR	0.24 (*P* < 0.01)	0.27 (*P* < 0.01)	0.25 (*P* < 0.01)	0.28 (*P* < 0.01)	0.23 (*P* < 0.01)
AGP	0.13 (*P* = 0.10)	0.17 (*P* = 0.03)	0.50 (*P* < 0.01)	–	0.68 (*P* < 0.01)
CRP	0.15 (*P* = 0.05)	0.18 (*P* = 0.02)	0.60 (*P* < 0.01)	0.68 (*P* < 0.01)	–

AGP = alpha-1-acid glycoprotein; CRP = C-reactive protein; IFN = interferon; IL = interleukin; KTR = kynurenine–tryptophan ratio.

**Table 4:** Linear regression models showing association of the measure of change in a biomarker (tryptophan, citrulline) by 1 SD on statural growth over the next 6 months that include age, LAZ at the time of biomarker assessment, and sex as covariates and a child-level random effect to account for repeated measures in subjects

		Change in LAZ 6 months after biomarker measure in Peru	Change in LAZ 6 months after biomarker measure in Tanzania	Combined change in LAZ 6 months after biomarker assessment
Tryptophan (per SD)		0.105 (0.047 to 0.162) (*P* < 0.001)	0.128 (0.032 to 0.223) (*P* = 0.009)	0.111 (0.061 to 0.162) (*P* < 0.001)
Age	7 months	Ref.	Ref.	Ref.
	15 months	0.102 (0.010 to 0.195) (*P* = 0.030)	0.307 (0.147 to 0.467) (*P* < 0.001)	0.171 (0.090 to 0.252) (*P* < 0.001)
	24 months	0.307 (0.180 to 0.434) (*P* < 0.001)	0.588 (0.267 to 0.909) (*P* < 0.001)	0.364 (0.240 to 0.487) (*P* < 0.001)
Gender		0.045 (−0.049 to 0.138) (*P* = 0.347)	0.178 (0.022 to 0.335) (*P* = 0.026)	0.106 (0.022 to 0.191) (*P* = 0.013)
Baseline LAZ		−0.200 (−0.249 to −0.150) (*P* < 0.001)	−0.233 (−0.314 to −0.152) (*P* < 0.001)	−0.232 (−0.276 to −0.188) (*P* < 0.001)
Country	Peru	NA	NA	−0.362 (−0.454, −0.270) (*P* < 0.001)
	Tanzania	NA	NA	
Constant		−0.899 (−1.140 to −0.659) (*P* < 0.001)	−1.681 (−2.053 to −1.310) (*P* < 0.001)	−1.095 (−1.310 to −0.880) (*P* < 0.001)

Citrulline (per SD)		−0.001 (−0.055 to 0.053) (*P* = 0.977)	−0.001 (−0.085 to 0.083) (*P* = 0.980)	
Age	7 months	Ref.	Ref.	
	15 months	0.077 (−0.022 to 0.175) (*P* = 0.128)	0.283 (0.116 to 0.450) (*P* = 0.001)	
	24 months	0.279 (0.119 to 0.439) (*P* = 0.001)	0.523 (0.191 to 0.855) (*P* = 0.002)	
Gender		0.037 (−0.057 to 0.131) (*P* = 0.438)	0.167 (0.010 to 0.325) (*P* = 0.038)	
Baseline LAZ		−0.189 (−0.239 to −0.139) (*P* < 0.001)	−0.210 (−0.291 to −0.129) (*P* < 0.001)	
Country	Peru	NA	NA	
	Tanzania	NA	NA	
Constant		−0.584 (−0.786 to −0.382) (*P* < 0.001)	−1.344 (−1.684 to −1.003) (*P* < 0.001)	

LAZ = length-for-age Z-score; NA = not applicable; SD = standard deviation.
